# Genome dynamics in major bacterial pathogens

**DOI:** 10.1111/j.1574-6976.2009.00173.x

**Published:** 2009-04

**Authors:** Ole Herman Ambur, Tonje Davidsen, Stephan A Frye, Seetha V Balasingham, Karin Lagesen, Torbjørn Rognes, Tone Tønjum

**Affiliations:** 1 Centre for Molecular Biology and Neuroscience, Institute of Microbiology, University of Oslo, Oslo University Hospital (Rikshospitalet)Oslo, Norway; 2 Centre for Molecular Biology and Neuroscience, Institute of Microbiology, University of OsloOslo, Norway; 3 Department of Informatics, University of OsloOslo, Norway

**Keywords:** genome sequences, gene profile, DNA repair, recombination, competence, transformation

## Abstract

Pathogenic bacteria continuously encounter multiple forms of stress in their hostile environments, which leads to DNA damage. With the new insight into biology offered by genome sequences, the elucidation of the gene content encoding proteins provides clues toward understanding the microbial lifestyle related to habitat and niche. *Campylobacter jejuni, Haemophilus influenzae, Helicobacter pylori, Mycobacterium tuberculosis*, the pathogenic *Neisseria, Streptococcus pneumoniae, Streptococcus pyogenes* and *Staphylococcus aureus* are major human pathogens causing detrimental morbidity and mortality at a global scale. An algorithm for the clustering of orthologs was established in order to identify whether orthologs of selected genes were present or absent in the genomes of the pathogenic bacteria under study. Based on the known genes for the various functions and their orthologs in selected pathogenic bacteria, an overview of the presence of the different types of genes was created. In this context, we focus on selected processes enabling genome dynamics in these particular pathogens, namely DNA repair, recombination and horizontal gene transfer. An understanding of the precise molecular functions of the enzymes participating in DNA metabolism and their importance in the maintenance of bacterial genome integrity has also, in recent years, indicated a future role for these enzymes as targets for therapeutic intervention.

## Introduction

Continuously, whole genome sequences of bacterial pathogens are being completed, allowing a comparative genomic analysis of the adaptation of different species to their natural habitats. We selected the genomes of nine pathogens and two model organisms for analysis of their gene complements related to genome maintenance and horizontal gene transfer (HGT). In this context, the aim was to focus on major pathogens with relatively small genomes exhibiting vivid genome dynamics, related to competence for transformation, and also include Gram-positive and Gram-negative representatives and model organisms, without covering a wall-to-wall panel for all infectious diseases. Among the major microbial pathogens dominating the human infectious disease scenario at the global level, *Neisseria meningitidis, Haemophilus influenzae* and *Streptococcus pneumoniae* are the causative agents of meningitis and airway-related infections. *Neisseria gonorrhoeae* is the causative agent of gonorrhoea, *Helicobacter pylori* is the cause of gastric and duodenal ulcers and precancerous gastric lesions, and *Campylobacter jejuni* is a main source of diarrhea. *Streptococcus pyogenes*, ‘the flesh-eating bug’ or group A streptococcus, is a major tissue destructor and the cause of a number of disease types including tonsillitis, serious skin infections with tissue damage, erysipelas, scarlatina, rheumatic fever and puerperal fever. *Staphylococcus aureus* is a typical abscess-forming agent including the methicillin-resistant *S. aureus* (MRSA), which is an emerging and feared multiresistant opportunist. *Mycobacterium tuberculosis* is the cause of tuberculosis, infecting one-third of the world's population, making it the most widespread pathogen known. Thus, *C. jejuni, H. influenzae, H. pylori, M. tuberculosis*, pathogenic *Neisseria, S. pneumoniae, S. pyogenes* and *S. aureus* all contribute to frequent infectious disease cases of mild to grave severity, as well as a large numbers of deaths each year. Most of these pathogens are opportunistic, mucosal surface or skin organisms, while *M. tuberculosis* is an intracellular parasite.

**Fig. 1:**
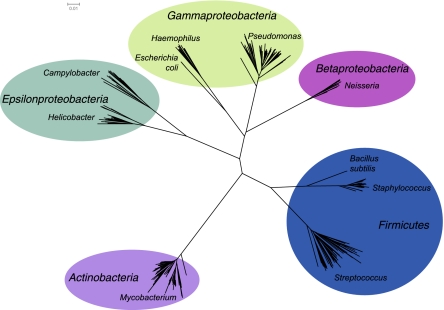
Phylogenetic map based on 16S rRNA gene sequences of the bacterial species under study.

Representing members of the phylae *Proteobacteriae, Actinobacteria* and *Firmicutes* (Fig. 1), each of these microbial pathogens exhibits a lifestyle and survival strategy relevant for their respective niches (Table 1): *N. meningitidis* (Parkhill *et al.*, 2000a; Tettelin *et al.*, 2000), *N. gonorrhoeae, H. influenzae* (Fleischmann *et al.*, 1995), *S. pneumoniae* (Tettelin *et al.*, 2001), *S. pyogenes* (Ferretti *et al.*, 2001; Hidalgo-Grass *et al.*, 2002), *C. jejuni* (Parkhill *et al.*, 2000b) and *H. pylori* (Alm *et al.*, 1999) are all fastidious and face up to their environments in their exclusive human host with small, but hyperdynamic genomes, while they are naturally competent for transformation. The genome of the versatile pathogen *S. pyogenes* contains all the predicted ORFs required to be competent for transformation (Ferretti *et al.*, 2001) and has also been shown to be competent (Hidalgo-Grass *et al.*, 2002). The noncompetent *M. tuberculosis* (Cole *et al.*, 1998) and *S. aureus* (Herron-Olson *et al.*, 2007) have larger genomes, reflecting their lifestyles and fitness for survival also in versatile environments outside the human body.

**Table 1:** Characteristics of major human pathogens and reference bacteria included in the study

Species (Classification) No. of genomes analyzed	Genome size (mb)	Habitat niche	Disease	References
*Neisseria meningitidis* (*Betaproteobacteria*) 5	2.2	Upper airway colonizer	Meningitis, septicemia, Waterhouse–Friderichsen syndrome	Parkhill *et al.* (2000a), Tettelin *et al.* (2000)
*Neisseria gonorrhoeae* (*Betaproteobacteria*) 1	2.1	Urethra	Gonorrhea	Oklahoma University Website
*Haemophilus influenzae* (*Gammaproteobacteria*) 6	1.9	Upper airway colonizer	Pneumonia, meningitis, bacteremia, otitis media and more	Fleischmann *et al.* (1995)
*Streptococcus pneumoniae* (*Bacilli*) 3	2.0	Upper airway colonizer	Pneumonia, septicemia, otitis media, peritonitis, pericarditis, meningitis, brain abscess and more	Tettelin *et al.* (2001)
*Helicobacter pylori* (*Epsilonproteobacteria*) 3	1.6	Stomach and duodenum	Peptic ulcer and gastric cancer	Alm *et al.* (1999)
*Campylobacter jejuni* (*Epsilonproteobacteria*) 6	1.6	Gastrointestinal	Main cause of diarrhea in Europe, inducer of the Guillain Barre syndrome	Parkhill *et al.* (2000b)
*Staphylococcus aureus*/MRSA (*Bacilli*) 14	2.8+	Nose and skin	Pneumonia, meningitis, toxic shock syndrome, septicemia and skin infections	Herron-Olson *et al.* (2007)
*Streptococcus pyogenes* (*Bacilli*) 14	1.9	Throat and skin	Diverse pathogenic outcomes/diseases – flesh-eating bacteria	Ferretti *et al.* (2001)
*M. tuberculosis* (*Actinobacteria*) 5	4.2+	Lungs, extrapulmonary locations	Tuberculosis	Cole *et al.* (1998)
*Bacillus subtilis* (*Bacilli*) 1	4.2	Soil	Reference bacterium	Kunst *et al.* (1997)
*Escherichia coli* (*Gammaproteobacteria*) 18	4.6+	Intestine, sewer	Reference bacterium	Blattner *et al.* (1997)

Here, we summarize comparative genomic characteristics of this subset of human pathogens and studies that have contributed to our understanding of how they adapt to different environments, combat antibiotics and acquire increased virulence. We address selected parts of the total gene content of these major pathogens in order to elucidate how these reflect their major traits and enable them to persist in their respective environments. As such, gene complements shed new light on the basis for genome dynamics in microbial pathogens. In the context of genome maintenance and HGT, major emphasis will be placed on DNA repair, type IV secretion and transformation processes.

## Methods

### Identification of orthologs by use of the DNA Repair Gene Orthologs system

Initially, genes known to be involved in different types of DNA repair, replication and recombination as well as genes responsible for secretion systems (mainly type II and IV secretion), pilus biogenesis and DNA uptake were identified in representative bacterial species on the basis of a combination of manual selection, databases (COG), lists in review papers (Cascales & Christie, 2004; Chen *et al.*, 2005) and other sources. A system for the identification of gene orthologs was then used in order to see whether orthologs of the selected genes were present or absent in the genomes of the selected pathogenic bacteria under the study listed in Table 1. Based on the known genes of the various functions and their orthologs in the selected pathogenic bacteria under study, an overview of the presence of the different types of genes was created.

In brief, the ortholog system DNA Repair Gene Orthologs system (T. Rognes, O. Aussedat & B. Eliassen, unpublished data) identifies orthologous genes based on similarity of sequence. Initially, all protein sequences in Refseq were compared using an all-vs.-all blast search, and all significant matches were identified (*E*<1e-7). Genes were linked using single-linkage clustering based on the protein sequence alignment scores, starting with the highest-scoring pairs of sequences and progressing to gradually lower-scoring pairs. Genes belonging to the same organisms were not allowed to be clustered, unless all genes in the cluster belonged to that same organism (inparalogs). Access to the clustering information was provided through a web interface, where organisms and groups of genes could be selected. In addition to DNA Repair Gene Orthologs, the KEGG pathway database (http://www.genome.jp/kegg/pathway.html) (Kanehisa *et al.*, 2008) was used for identifying orthologs. Positive hits, and lack thereof, were checked against homology identifications from http://www.microbesonline.org/ (Alm *et al.*, 2005) and blast searches.

## DNA repair and recombination

### DNA repair

**Fig. 2:**
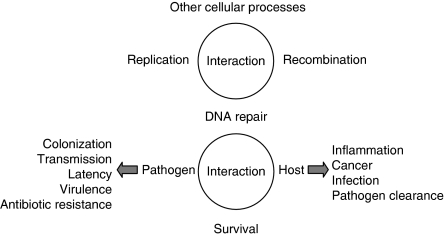
DNA repair, recombination and replication (3R) are essential processes in living cells. These processes are interconnected, often sharing components to restore or replicate genetic information. A growing body of evidence is pointing at the necessity of harboring 3R mechanisms for pathogens to effectively colonize their human host, which exerts, among others, oxidative stress on the bacterial genomes through the oxidative burst (described in the main text). Also, the host relies on 3R mechanisms to survive an invasion of potentially deadly organisms. Bacteria or bacterial components, as well as the inflammation process triggered by the bacteria, may induce host DNA damage (Box 1). The outcome of a bacterial invasion depends on both the host and the pathogen: they are not static players independent of each other. This interaction is best described as an interplay where the actions of one affects the other, for better or for worse. One example illustrating this scenario is the effect of antibiotics; although helping the host to clear the invading pathogen, induction of bacterial DNA repair mechanisms triggered by the antibiotic may lead to the dissemination of bacterial virulence determinants (Box 2).

DNA repair is essential to all organisms (Fig. 2). In the context of this review, we will focus particularly on the DNA repair machinery of the pathogens *C. jejuni, H. influenzae, H. pylori, M. tuberculosis*, the pathogenic *Neisseria, S. pneumoniae, S. pyogenes* and *S. aureus*, and how DNA repair contributes to the ability of these bacteria to colonize, transmit and survive inside their host.

**Box 1 Host DNA repair induced by pathogens**

DNA repair is critical for the survival of pathogens inside the host because of the DNA lesions introduced in the genome of the pathogen by harmful agents released from the host (e.g. through the oxidative burst). What happens in the host, related to DNA repair, when colonized by a pathogen? Recent literature point at several mechanisms for host DNA damage during pathogen colonization (this list is not exhaustive):

1. *Factors released from the bacteria cause DNA damage*: *Campylobacter jejuni* cytolethal distending toxin (CDT), a protein toxin affecting the cell cycle of the host, can induce DNA damage responses in human cells. This has been identified by the presence of Rad50 foci near sites of DNA damage and the formation of the phosphoproteinγ-H2AX that mediates recruitment of repair complexes to sites where double-strand breaks occur (Hassane *et al.*, 2003).
2. *Reactive oxygen species (ROS) induce DNA damage*: During chronic infection, ROS produced by factors of the host immune system can directly induce host DNA damage as has been shown by Obst *et al.* (2000). After incubation of gastric cells with *Helicobacter pylori* extracts, an induced synthesis of ROS and DNA fragmentation in host cells were identified.
3. *Host levels of DNA repair enzymes are altered*: Yao *et al.* (2006) recently demonstrated a reduction of mismatch repair protein levels in cells cocultured with *H. pylori* compared with cells not cocultured with this pathogen. The reduction of repair protein levels was associated with an increased number of frameshift and point mutations in the cells cocultured with *H. pylori*.
4. *Host DNA repair components are selectively mutated*: Shibata *et al.* (2002) found that *H. pylori* infection with strains harboring the pathogenicity island *cag* is associated with a higher prevalence of *p53* mutation in gastric adenocarcinoma.

**Box 2 Unintended consequences of antibiotic treatment**

Antibiotic treatment is the major ingredient in the battle against pathogenic bacteria. Resistance against such drugs has been a major concern since penicillin resistance was first discovered in *Staphylococcus aureus* in 1947. However, other important unintended side effects in the microorganisms have recently been described: several antimicrobial agents induce bacterial DNA responses, like the SOS system. In *S. aureus*, this induction results in replication and high-frequency transfer of a pathogenicity island, thereby promoting the spread of staphylococci virulence factors (Ubeda *et al.*, 2005). In bacteria not harboring an SOS system, like *Streptococcus pneumoniae*, antibiotic stress has been shown to induce genetic exchange through transformation (Prudhomme *et al.*, 2006), a mechanism that may contribute to the acquisition of new survival or virulence determinants.

Nearly 60 years ago, the studies of *Escherichia coli* DNA repair systems were initiated (Friedberg, 2008), and this organism now represents the most well-characterized bacterium of all. However, there is a growing body of evidence showing that not all bacteria function as *E. coli*, and in order to gain a wider genome-based perspective beyond the *E. coli* paradigm, a thorough analysis of different groups of bacteria is warranted. *Campylobacter jejuni, H. influenzae, H. pylori, M. tuberculosis*, pathogenic *Neisseria, S. pneumoniae, S. pyogenes* and *S. aureus* contribute to the majority of morbidity and mortality caused by bacteria worldwide. At the same time, *E. coli* and *Bacillus subtilis* serve as Gram-negative and Gram-positive model organisms, respectively. When comparing the DNA repair, recombination and replication (3R) enzymes in these bacteria with those present in *E. coli* (Table 2), a general theme seems to be the occurrence of a reduced number of genes in each class of DNA repair as compared with *E. coli*: in base excision repair, which normally removes subtle base damages (Seeberg *et al.*, 1995), *nei, alkA, nfi, nfo, tag* and *xthA* are often not present in these pathogens. In the postreplication mismatch repair (MMR) pathway, base–base mismatches and insertion/deletion loops (IDLs) are recognized and excised (Schofield & Hsieh, 2003). The key enzymes of this pathway, MutS and MutL, are absent in some of the pathogens, as is MutH. A more detailed description of this pathway and some interesting features are given below. Direct repair, in which DNA lesions are chemically reversed (Mishina *et al.*, 2006), and especially for enzymes handling alkylation damage (*ada* and *alkB*), the pathogens often show an absence of genes. On the one hand, the apparent lack of function might allow adapted genome dynamics. On the other hand, one needs to bear in mind that there might exist genes encoding products that perform identical functions, but that lack sequence homology. In this context, new protein-encoding and RNA genes remain to be discovered. Also, a number of (error-prone) DNA polymerases and the SOS response regulator, LexA, are often not present in these bacteria. Likewise, when considering helicases, which are important proteins involved in various aspects of 3R activities, *E. coli* is the organism studied most and is generously equipped (Table 3). The only 3R pathways that appear to be ubiquitous for most organisms are nucleotide excision repair, recombinational repair and replication (Table 3). Nucleotide excision repair removes many types of bulky lesions from DNA, often of exogenous origin, while recombinational repair is crucial for the repair of DNA strand breaks that occur during recombination (de Laat *et al.*, 1999). Replication is fundamental for the perpetuation of the genome, and this process is tightly coupled to most of the DNA repair pathways (Friedberg *et al.*, 2006). Our observations (Table 3) corroborate the results of Eisen & Hanawalt (1999), who performed a phylogenomic study of DNA repair genes, proteins and processes in, among others, 11 bacterial species. An immediate question arising from these findings is that how representative for each species is the number of DNA repair genes found in one strain, considering the variable level conservation and diversity in clonal and polyphyletic species? In a larger context, more central questions arising from the discussion above are as follows: how does the DNA repair enzyme repertoire affect the lifestyle of the bacteria? For instance, what does it mean for *Neisseria* sp. not to host an SOS response? Or for *M. tuberculosis* not to encode conventional mismatch repair? How do DNA repair enzymes from different pathways interact? How do DNA repair enzymes interact with enzymes from other cellular systems? And most importantly, how are colonization, transmission and virulence of the pathogenic bacteria affected by the presence or the absence of specific DNA repair enzymes?

**Table 3:** Distribution of helicases involved in DNA repair, replication and recombination among the bacteria examined

Symbol	Description	*B.s.*	*E.c*.	*C.j*.	*H.i.*	*H.p.*	*N.g.*	*N.m.*	*M.t.*	*S.a.*	*S.p.*	*S.py.*
*dnaB*	Replicative DNA helicase	^*^	^*^	^*^	^*^	^*^	^*^	^*^	^*^	^*^	^*^	^*^
*uvrD*	DNA-dependent ATPase I and helicase II	^*^	^*^	^*^	^*^	^*^	^*^	^*^	^**^	^*^	^*^	^*^
*dinG*	ATP-dependent DNA helicase	^*^	^*^				^*^	^*^	^*^		^*^	^*^
*lhr*	Predicted ATP-dependent helicase		^*^						^*^			
*recG*	ATP-dependent DNA helicase	^*^	^*^	^*^	^*^	^*^	^*^	^*^	^*^	^*^	^*^	^*^
*recQ*	ATP-dependent DNA helicase	^*^	^*^		^*^		^*^	^*^		^*^		
*ruvB*	ATP-dependent DNA helicase, component of RuvABC resolvasome	^*^	^*^	^*^	^*^	^*^	^*^	^*^	^*^	^*^	^*^	^*^
*ruvA*	Component of RuvABC resolvasome, regulatory subunit	^*^	^*^	^*^	^*^	^*^	^*^	^*^	^*^	^*^	^*^	^*^
*ercc3*	Excision repair cross-complementing rodent repair								^*^			
*mfd*	Transcription-repair coupling factor	^*^	^*^	^*^	^*^	^*^	^*^	^*^	^*^	^*^	^*^	^*^

*B.s., Bacillus subtilis*; *E.c., Escherichia coli*; *C.j., Campylobacter jejuni*; *H.i., Haemophilus influenzae*; *H.p., Helicobacter pylori*; *N.g., Neisseria gonorrhoea*; *N.m., Neisseria meningitidis*; *M.t., Mycobacterium tuberculosis*; *S.a., Staphylococcus aureus*; *S.p., Streptococcus pneumoniae*; *S.py., Streptococcus pyogenes*.

**Table 2:** Selected genes in major DNA repair pathways, recombination and replication that are present in the genome sequences of the human pathogens *Campylobacter jejuni* NCTC 11168 (*C.j.*), *Haemophilus influenzae* Rd KW20 (*H.i*.), *Helicobacter pylori* J99 (*H.p*.), *Neisseria gonorrhoeae* FA1090 (*N.g*.), *Neisseriameningitidis* MC58 (*N.m*.), *Mycobacterium tuberculosis* H37Rv (*M.t.*), *Staphylococcusaureus* MRSA 252 (*S.a*.), *Streptococcuspneumoniae* TIGR4 (*S.p*.) and *Streptococcuspyogenes* SF370 (*S.py*.) and reference bacteria *Bacillus subtilis* ssp. subtilis str. 168 (*B.s*.) and *Escherichia coli* K12 (*E.c*.)

Symbol	Description	*B.s.*	*E.c.*	*C.j.*	*H.i.*	*H.p.*	*N.g.*	*N.m.*	*M.t.*	*S.a.*	*S.p.*	*S.py.*
Direct repair†												
*ada*	Methyltransferase, transcriptional regulator	*	*	*					*			
*alkB*	Oxidative demethylase		*						*			
*ogt*	Methyltransferase	*	*			*			*		*	
*phr/spl*	Photolyase	*	*				*	*		*		*
Base excision repair†												
*mutY*	Adenine DNA glycosylase	*	*	*	*	*	*	*	*	*	*	*
*mutM*	Formamidopyrimidine DNA glycosylase	*	*		*		*	*	*	*	*	*
*nei*	Endonuclease VIII		*						*			
*nth*	Endonuclease III	*	*	*	*	*	*	*	*	*	*	*
*tag*	3-Methyl-adenine DNA glycosylase I		*		*		*	*	*	*	*	
*alkA*	3-Methyl-adenine DNA glycosylase II		*						*			
*ung*	Uracil-DNA glycosylase	*	*	*	*	*	*	*	*	*	*	*
*xth*	Exonuclease III	*	*	*	*		*	*	*			*
*mpg*	3-Methylpurine DNA glycosylase	*							*	*		
*ygjF*	Thymine-DNA-glycosylase		*									
*nfo*	Endonuclease IV	*	*							*		
Nucleotide excision repair†												
*uvrA*	Damage recognition, ATPase	*	*	*	*	*	*	*	*	*	*	*
*uvrB*	Exinuclease	*	*	*	*	*	*	*	*	*	*	*
*uvrC*	Exinuclease	*	*	*	*	*	*	*	*	*	*	*
*uvrD*	Helicase II, DNA-dependent ATPase	*	*	*	*	*	*	*	*	*	*	*
*mfd*	Transcription-repair coupling factor	*	*	*	*	*	*	*	*	*	*	*
Mismatch repair†												
*mutS*	MutS1, mismatch recognition	*	*		*		*	*		*	*	*
*mutL*	Endonuclease/recruitment of MutS	*	*		*		*	*		*	*	*
*mutH*	Endonuclease		*		*							
Recombinational repair†												
*recA*	DNA strand exchange and recombination protein	*	*	*	*	*	*	*	*	*	*	*
*recB*	Exonuclease V, β subunit		*		*		*	*	*			
*recC*	Exonuclease V, γ chain		*		*		*	*	*			
*recD*	Exonuclease V, α chain		*		*		*	*	*			
*recF*	Gap repair protein	*	*		*				*	*	*	*
*recO*	Gap repair protein	*	*		*		*	*	*	*	*	*
*recR*	Gap repair protein	*	*	*	*	*	*	*	*	*	*	*
*ruvA*	RuvABC resolvasome, regulatory subunit	*	*	*	*	*	*	*	*	*	*	*
*ruvB*	RuvABC resolvasome, DNA helicase	*	*	*	*	*	*	*	*	*	*	*
*ruvC/recU*	RuvABC resolvasome, endonuclease	*	*	*	*	*	*	*	*			
*ssb*	Single-stranded-binding protein	*	*	*	*	*	*	*	*		*	*
Other repair†												
*lexA*	Transcriptional repressor of SOS regulon	*	*		*				*	*		
*polB*	DNA polymerase II		*									
*umuC*	DNA polymerase V, subunit C	*	*									
*umuD*	DNA polymerase V, subunit D		*									
*dinB*	DNA polymerase IV	*	*		*		*	*	*	*	*	*
*ligA*	DNA ligase	*	*	*	*	*	*	*	*	*	*	*
*mutT*	Pyrophosphohydrolase		*		*		*	*	*			
Replication†												
*dnaA*	Chromosomal replication initiator protein	*	*	*	*	*	*	*	*	*	*	*
*dnaB*	Replicative DNA helicase	*	*	*	*	*	*	*	*	*	*	*
*dnaG*	DNA primase	*	*	*	*	*	*	*	*	*	*	*
*gyrA*	DNA gyrase, subunit A	*	*	*	*	*	*	*	*	*	*	*
*gyrB*	DNA gyrase, subunit B	*	*	*	*	*	*	*	*	*	*	*
*parC*	DNA topoisomerase IV, subunit A	*	*		*		*	*		*	*	*
*parE*	DNA topoisomerase IV, subunit B	*	*		*		*	*		*	*	*
*priA*	Primosome assembly protein	*	*	*	*	*	*	*	*	*	*	*
*rep*	DNA helicase	*	*	*	*	*	*	*				
*topA*	DNA topoisomerase I	*	*	*	*	*	*	*	*	*	*	*
*polA*	DNA polymerase I	*	*	*	*	*	*	*	*	*	*	*

A complete list of the genes associated with DNA repair, recombination and replication can be found at http://cmr.jcvi.org/cgi-bin/CMR/shared/Genomes.cgi.

* At least one sequence homolog is present; whether the gene contains point mutations and authentic frameshifts or whether the enzyme is active or not is not considered.

† Results based on the presence of genes as identified through the DNA Repair Gene orthologs database (T. Rognes *et al.*, unpublished data) and TIGR genome sequences, role category: DNA metabolism, DNA replication, recombination and repair (http://cmr.jcvi.org/cgi-bin/CMR/shared/Genomes.cgi).

Although substantial wet-lab analysis regarding DNA repair enzymes in these pathogens is not available, some studies have been conducted. The readers are referred to recent summaries (Davidsen & Tønjum, 2006; Wang *et al.*, 2006; Davidsen *et al.*, 2007a) and the present review (dos Vultos *et al.*, 2009) on DNA repair in *N. meningitidis, H. influenzae, S. pneumoniae, H. pylori* and *M. tuberculosis*. For instance, in *N. meningitidis* mutants inactivated in genes representing all the main DNA repair pathways, Davidsen *et al.* (2007b) have demonstrated that the highest spontaneous mutation frequency among the *N. meningitidis* single mutants are found in MutY-deficient strains, as opposed to *mutS* mutants in *E. coli*, indicating a possible role for meningococcal MutY in antibiotic resistance development. In general, distinct differences between *N. meningitidis* and established DNA repair characteristics in *E. coli* have been found. Interestingly, an increasing number of studies are focusing on the *in vivo* survival of DNA repair mutants in animal models. In *M. tuberculosis*, it has been shown that a nucleotide excision repair mutant and a DNA polymerase E2 mutant are attenuated in mice (Boshoff *et al.*, 2003; Darwin & Nathan, 2005). In *H. pylori*, the base excision glycosylases MutY and Nth, as well as recombinational repair, are required for effective colonization of the stomach of mice (O'Rourke *et al.*, 2003; Eutsey *et al.*, 2007; Amundsen *et al.*, 2008; Wang & Maier, 2008). These findings suggest that the host induces DNA lesions in the genomes of the infectious agents, and therefore effective DNA repair is crucial for the pathogen to be able to colonize its host (Fig. 2). The observations are also supported by gene expression analysis of the pathogens upon contact with host cells. In several studies, DNA repair components have been found to be upregulated upon interaction with human cells (Vriesema *et al.*, 2000; Morelle *et al.*, 2005; Tala *et al.*, 2008). In contrast to the importance of functional DNA repair mechanisms for effective host colonization stands the putative correlation between the lack of DNA repair genes associated with transmission and virulence of the pathogens. *Neisseria meningitidis* strains harboring mutated *mutS* or *mutL* gene copies have been identified at high prevalence in epidemic serogroup A isolates, while *mutT* and *ogt* mutations have been characterized in strains belonging to the hypervirulent *M. tuberculosis* W-Beijing family (Richardson *et al.*, 2002; Rad *et al.*, 2003). Such DNA repair deficiencies are often accompanied by a hypermutator phenotype that may be beneficial under specific selective pressures (Giraud *et al.*, 2001). In a clinical setting, this is highly relevant concerning the development of antibiotic resistance. In *S. pneumoniae*, MMR mutants may have a selective advantage in the setting of antibiotic pressure (Gould *et al.*, 2007), while Schaaff *et al.* (2002) recently showed that an elevated mutation frequency favors development of vancomycin resistance in *S. aureus*. Likewise, high mutation frequencies have been suggested as the cause of the frequently acquired antibiotic resistance during treatment of *H. pylori* infections (Bjorkholm *et al.*, 2001). However, a general role for hypermutators in the emergence of clinically relevant antibiotic resistance and disease remains to be elucidated (Woodford & Ellington, 2007). Thus, the absence of certain DNA repair activities may be beneficial and allow adaptation during specific stages of the pathogen's life cycle, but intact repair machineries are vital for long-term colonization. This scenario highlights the importance for bacteria to possess mechanisms for reacquiring genes encoding DNA repair functions at some stage, for example through HGT.

### Focus on MMR

#### MMR functions

The DNA MMR pathway is conserved from prokaryotes/bacteria to eukaryotes including humans. Defects in MMR increase mutation rates and cause genome instability, which in turn may expand the fitness landscape of bacterial pathogens. In humans, impaired MMR may cause a range of cancers, and the documented association to hereditary nonpolyposis colorectal cancer (or Lynch syndrome) has been studied intensively (Lynch & Lynch, 1985). MMR is a postreplicative process and provides an efficient way of repairing both base mismatches and IDLs that are generated during DNA synthesis. In essence, MMR allows degradation of error-containing DNA and resynthesis of unimpaired DNA. High-fidelity DNA replication is central to genome maintenance, and evidence for a close spatio-temporal association between replication, recombination and MMR is growing (Simmons *et al.*, 2008).

MMR has been extensively investigated in *E. coli* and much of our current knowledge is obtained from studies of this model organism. In short, the process of MMR can be summarized as follows: MutS binds the mismatch and recruits MutL, which orchestrates several interactions including the activation of MutH – an endonuclease nicking the unmethylated strand of newly synthesized DNA at GATC sites. A piece of the nascent DNA that has received a nick is degraded by exonucleases with the aid of the DNA helicase UvrD before DNA polymerase III accurately resynthesizes DNA, and the remaining nick is sealed by DNA ligase. The process also depends on single-strand-binding proteins and the initial methylation of GATC sites by Dam methylase. The strand containing the mismatch is degraded in the 5′-to-3′ or 3′-to-5′ direction, depending on the location of the mismatch relative to the nick. The minimal human MMR has also been reconstituted *in vitro* by the following elements: MutSα, MutLα, ExoI, proliferating cell nuclear antigen (PCNA), replication factor C (which loads PCNA onto DNA), the single-strand-binding factor replication protein A, polδ and DNA ligase I (Constantin *et al.*, 2005; Zhang *et al.*, 2005). It is now clear, however, that many bacteria differ from *E. coli* in their basic MMR machinery.

#### The conundrum of MutH vs. MutL

The absence of MutH in many bacteria and all eukaryotes is particularly striking, because it suggests that strand discrimination and the initiation of excision may have a basis different from that of the methyl-directed process in *E. coli* and in certain other Gram-negative microorganisms. How can MMR discriminate between the template strand and the newly synthesized strand if it is not methyl-directed? It has been proposed that MMR can be directed to the newly synthesized strand by interacting with strand termini during replication. As such, MMR would constitute a part of the replisome and could direct repair activity from the termini between okazaki fragments on the lagging strand or from the 3′-terminus on the leading strand, linking the two processes tightly (Jiricny, 2006). Indeed, the interaction between MutS from *B. subtilis*, which does not belong to the methyl-directed MMR group, and the β-clamp (PCNA in eukaryotes) was recently described in detail and supported that the MMR complex acts at, or in association with, the replication fork (Simmons *et al.*, 2008). A breakthrough came from the laboratory of Paul Modrich when they found that human MutLα itself is an endonuclease that is able to produce a nick in nascent DNA (Kadyrov *et al.*, 2006). A model where MutL can introduce random nicks on both sides of the mismatch has helped explain how a bidirectional DNA repair process was able to operate with a single exonuclease (EXOI in the human model) that degrades only in the 5′-3′ direction. Based on the finding that endonucleolytic hydrolysis of DNA depends on one or two divalent cations as the metal required, a binding site with a motif [DQHA(X)_2_E(X)_4_E] was identified, which is conserved in archaeal, eukaryotic (PMS2 and MLH3) and eubacterial MutL homologs. Convincingly, this motif was absent in all MutL homologs from those Gram-negative organisms known to have MutH and the methyl-directed MMR pathway (Kadyrov *et al.*, 2006). We compiled a list of organisms containing a MutH homolog (Supporting Information, Table S1) and found that the distribution of MutH-dependent MMR is very limited. With a few exceptions, MutH is primarily found in bacterial species sorting under the class of *Gammaproteobacteria*. In our selection of pathogens, only *H. influenzae* belonging to the *Pasteurellaceae* (class *Gammaproteobacteria*) contains the *mutH* gene whereas the *Neisseria* (class *Betaproteobacteria*) and the staphylococci and streptococci (both class *Bacilli*) contain the MutH-less MMR (Table 2 and Fig. 1). The *Epsilonproteobacteria* member *C. jejuni* and the Actinobacterium *M. tuberculosis* lack MMR altogether. *Helicobacter pylori* contains a gene encoding a homolog of MutS-2, but this protein is likely to be involved in processes other than MMR as we know it (Table 2). Taken together, these bacteria may experience elevated mutation rates because they lack a functional MMR system.

An alignment based on the MutL pfam entries from 822 organisms was used to investigate sequence conservation of the metal-binding motif. We note that the motif in *Neisseria* sp. has a Q/M substitution in the second position DQ/MHA(X)_2_E(X)_4_E. As Fig. S1 shows, the Q/M substitution is one of the most common substitutions. Whether this substitution from a polar to a nonpolar amino acid is functionally important for MutL activity and function remains to be investigated. It has, however, been established that MutL knock-outs in *N. meningitidis* produce a mutator phenotype as expected for MMR malfunction (Richardson *et al.*, 2002).

#### MutS sequence diversity

Genome comparisons have also revealed differences in the distribution of the *mutS* gene and also great diversity within the *mutS*-group. Phylogenomic analysis has revealed that the *mutS* lineage split early in evolution and gave several distinct lines, where, importantly, only one belongs to the MMR pathway (Eisen, 1998; Lin *et al.*, 2007).

#### When MMR is absent

One of the striking characteristics of the *M. tuberculosis* and partially for *H. pylori* DNA repair system is the absence of recognized MMR homologs, which might suggest that these bacteria do not perform MMR activity. The consequent reduced fidelity in genome maintenance might add to the adaptive ability of *M. tuberculosis*, which otherwise seems to exist in genetic isolation. On the other hand, MMR activity could exist without sequence homology to recognized MMR components, and the search for components exerting MMR activity in *M. tuberculosis* should still be pursued.

### Distribution of helicases in pathogenic bacteria

Helicases are ubiquitous enzymes vital to all living organisms. They are motor proteins that move directionally along the nucleic acid phosphodiester backbone separating two annealed nucleic acid strands using energy from NTP hydrolysis. Helicases are involved in various aspects of cellular processes including replication, repair, recombination, transcription and RNA processing (Schmid & Linder, 1992; Matson *et al.*, 1994). The vital role(s) that these enzymes play has been underscored by a number of genetic discoveries. Mutations in three out of the five human *recQ* homologs have been identified as causes of Werner (WRN), Bloom (BLM) or Rothmund–Thomson syndrome (RECQ4), respectively (Ellis *et al.*, 1995; Yu *et al.*, 1996; Kitao *et al.*, 1999). Mutations interfering with the proper function of XPB and XPD helicases in humans have been linked to disorders such as Xeroderma Pigmentosum (XP), Cockayne syndrome (CS) and trichothiodystrophy (TTD) (Hoeijmakers, 1994; Vermeulen *et al.*, 1994; de Boer & Hoeijmakers, 2000).

First discovered in *E. coli* as a ‘DNA-unwinding enzyme’ more than 32 years ago, the number of helicases identified and characterized has since then increased tremendously (Abdel-Monem & Hoffmann-Berling, 1976). Most organisms host multiple helicases; for example the *E. coli* genome encodes at least 12 helicases (Matson *et al.*, 1994). When examining the pathogens under study (Table 3), some helicases that are essential to cellular functions, such as DnaB and UvrD, are distributed across all the organisms. In addition, RecG, RuvA and RuvB helicases that participate in recombinational repair, as well as Mfd involved in nucleotide excision repair, are found in all the pathogens. On the other hand, some helicases, such as RecQ, Ercc3, DinG and Lhr, are not universally distributed in our selected organisms (Table 3). The *recQ* gene homolog is present in *H. influenzae*, the *Neisseria* and *S. aureus* while it is missing in *C. jejuni, H. pylori, M. tuberculosis, S. pneumoniae* and *S. pyogenes*. The *E. coli* RecQ DNA helicase has served as a paradigm for the RecQ family and has been proposed to have multiple functions in the initiation of recombination, resolution of recombination intermediates and suppression of illegitimate recombination also required for proper induction of the ‘SOS’ response to stalled replication forks (Hishida *et al.*, 2004; Chow & Courcelle, 2007).

The helicase- and RNAse-like C-terminal (HRDC) domain is characteristic of many members of the RecQ helicase clade (Bernstein & Keck, 2005; Wu *et al.*, 2005; Killoran & Keck, 2006a). Interestingly, RecQ, which usually contains a single HRDC domain in most organisms, is identified with three HRDC domains in *N. meningitidis* and *N. gonorrhoeae* and plays a critical role in determining pilin antigenic variation and also participates in DNA repair (Mehr & Seifert, 1998; Killoran & Keck, 2006b, 2008; Stohl & Seifert, 2006). This might indicate that the multiplicity of HRDC domains can represent one specialized way to exert specificity in RecQ activities (Killoran *et al.*, 2009). Even though RecQ is absent in *M. tuberculosis*, the HRDC domain is identified in its UvrD2 helicase, which is one out of the two UvrD-like paralogs found in mycobacteria (Morozov *et al.*, 1997; Sinha *et al.*, 2008). However, the HRDC domain appeared not to be essential for enzymatic activity of UvrD2, suggesting that it might be involved in DNA binding (Sinha *et al.*, 2008). It was proposed that the HRDC domain might target RecQ-family proteins to specific DNA structures (Bernstein & Keck, 2005).

Another feature noted among the distribution of the helicases in the pathogenic bacteria under study is the presence of XPB/ERCC3 homolog, which is found only in *M. tuberculosis* (Table 3) (Poterszman *et al.*, 1997). XPB/ERCC3/RAD25 in eukaryotes is an integral subunit of the transcription factor TFIIH, which is involved in transcription initiation and nucleotide excision repair (Weeda *et al.*, 1990; Schaeffer *et al.*, 1993). Even though well studied in humans, the role of ERCC3 helicase in bacteria is not yet known. However, the occurrence of the *ercc3* gene in prokaryotes seems to be limited to mycobacteria and *Kineococcus radiotolerans* (Biswas *et al.*, 2009), which might have acquired the gene through infrequent HGT that might occur from eukaryotes to certain bacterial species (Poterszman *et al.*, 1997; Aravind *et al.*, 1999).

The DinG helicase in *E. coli* is a damage-inducible, SOS-regulated, strucure-specific enzyme, related to the human helicases XPD and BACH1, Rad3 from *Saccharomyces cerevisiae* and Rad15 from *Schizosaccharomyces pombe* (see Voloshin & Camerini-Otero, 2007 and references therein) (Voloshin & Camerini-Otero, 2007). Similar to XPB, XPD is also a part of the multisubunit complex TFIIH that plays a dual role in the transcription initiation and nucleotide excision repair (de Boer & Hoeijmakers, 2000).

Another less-distributed helicase, the long helicase-related protein (Lhr), which is the longest protein identified in *E. coli*, is also found in *M. tuberculosis* (Table 3) (Reuven *et al.*, 1995). However, its exact function is not yet known. The fact that the *M. tuberculosis* genome encodes the eukaryotic DNA repair proteins such as ERCC3 and Mpg might reflect past HGT events and enable this bacterium to survive in the hostile environment inside human macrophages.

## HGT

### Natural transformation in selected pathogens

#### Transformation, type IV pili and type II and IV secretions

**Fig. 3:**
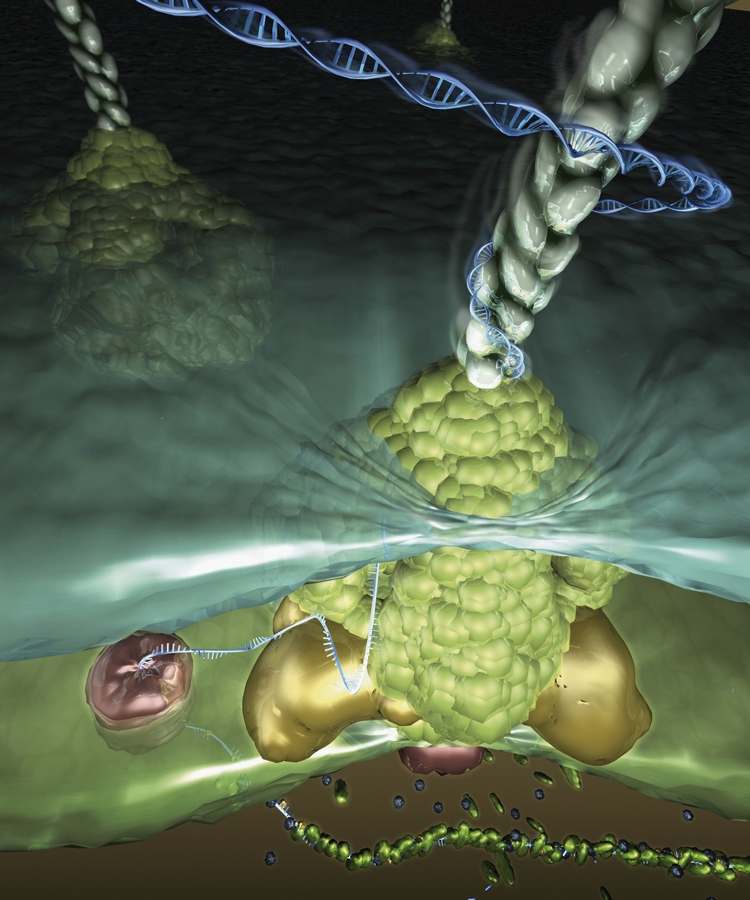
Model of the meningococcal transformation machinery based on the current information on the components involved in this process. DNA is predicted to enter the meningococcal cell through the PilQ pore, which, when it is wound around the pilus rod, sterically just allows the DNA to enter the cell. This hypothesis needs to be biologically verified.

The strong selective advantage of HGT has in several instances driven the evolution of complex machineries in favor of transformation. These divergent competence vehicles can ultimately cause the acquisition of novel traits such as antibiotic resistance, while they still allow homologous recombination that in turn could facilitate DNA repair and the fixation of beneficial alleles. Because different bacteria have solved their sex drives in various ways, the study of their strategies provides an exemplary case of convergent evolution. An interesting feature of all these systems is that they are based on structures already present in the cell that have become modified to facilitate and control genetic flux. *Neisseria meningitidis, H. influenzae* and the two streptococcal species *S. pneumoniae* and *S. pyogenes* all host systems composed of partners involved in the assembly of type IV pili and proteins with homology to type II secretion systems (Table 4) (Woodbury *et al.*, 2006). Type IV pili are important virulence factors in many pathogens, are required for transformation and are also associated with many other functions including cell adhesion, twitching motility and biofilm formation (Tønjum & Koomey, 1997; Mattick, 2002). In order to identify the entire complement of proteins driving the transformation machinery, the complete set of neisserial DNA-binding proteins should be defined (Lång *et al.*, 2009) (Fig. 3). Interestingly, the pilus biogenesis component PilQ binds DNA (Assalkhou *et al.*, 2007), thus contributing to the transformation process directly and indirectly, through pilus biogenesis. Type II secretion is also important for pathogenesis in facilitating the release of toxins and hydrolytic enzymes (Sandkvist, 2001). *Helicobacter pylori* and *C. jejuni* make use of a different transformation system that is evolutionarily related to type IV secretion systems. This is a secretion system that runs in reverse and resembles the conjugation systems of *Agrobacterium tumefaciens* (see separate section). *Staphylococcus aureus*, including MRSA, are not competent for transformation, but do contain a few of the known competence genes (ComC, ComGA, ComGB, ComGC) (Table 4). These are likely to be involved in other transport processes in this organism (Sibbald *et al.*, 2006). Strains of *S. aureus* display up to 20% variability in their genome sequence, and virulence and evolution of *S. aureus* are influenced by frequently occurring prophages and pathogenicity islands. It seems that prophages in moderately virulent *S. aureus* strains contribute important properties to pathogenesis, as fewer virulence factors in these cases are found outside of the prophages than for the highly virulent strains identified (Baba *et al.*, 2008). Similarly, *M. tuberculosis* is nontransformable and nonconjugative, although (remnants of) a few transducing phages are found. Despite its relative genetic/sexual isolation, this intensively studied organism is a most successful pathogen, and ends up with a clonal lifestyle (for the 3R and recombinational history of *Mycobacterium*, see review dos Vultos, in this issue; Jang *et al.*, 2008; Stinear *et al.*, 2008).

**Table 4:** The competence gene profile and presence and absence of genes related to competence in major microbial pathogens and reference bacteria

		Organism										
		*B.s*.	*E.c*	*C.j*.	*H.i*.	*H.p*.	*N.g*	*N.m*	*M.t*	*S.a*	*S.p*.	*S.py*.
Symbol	Description/function	G+	G−	G−	G−	G−	G−	G−	G+	G+	G+	G+
Competence/type II secretion/pilus biogenesis proteins												
ComEA	Competence protein, helix–hairpin–helix region	^*^	^*^	^*^	^*^		^*^	^*^	^*^	^*^	^*^	^*^
ComEC	Hydrolase, Rec2	^*^	^*^	^*^	^*^	^*^	^*^	^*^	^*^	^*^	^*^	^*^
ComFA	Late competence protein	^*^								^*^	^*^	^*^
ComFB	Competence protein FB	^*^										
ComFC	Amidophosphoribosyltransferase,	^*^	^*^	^*^	^*^	^*^	^*^	^*^	^*^	^*^	^*^	^*^
ComGA	ATPase, PulE/PilB/PilF/PilT/PilU	^*^	^*^	^*^	^*^	^*^	^*^	^*^		^*^	^*^	^*^
ComGB	Type II secretory pathway, PulF/PilG	^*^	^*^	^*^	^*^		^*^	^*^		^*^	^*^	^*^
ComGC-GG	Pilin/pseudopilin, PilA, PilE	^*^	^*^	^*^	^*^		^*^	^*^		^*^	^*^	^*^
ComC	Type IV prepilin peptidase, PilD	^*^	^*^	^*^	^*^		^*^	^*^		^*^	^*^	^*^
Smf	DNA-processing chain A, DprA	^*^	^*^	^*^	^*^	^*^	^*^	^*^	^*^	^*^	^*^	^*^
CinA	Competence/damage-inducible protein	^*^	^*^	^*^		^*^	^*^	^*^	^*^	^*^	^*^	^*^
ComL	Competence lipoprotein		^*^	^*^	^*^	^*^	^*^	^*^	^*^			
PilQ	Secretin, HofQ, ComE, PulD		^*^	^*^	^*^		^*^	^*^				

*B.s., Bacillus subtilis*; *E.c., Escherichia coli*; *C.j., Campylobacter jejuni*; *H.i., Haemophilus influenzae*; *H.p., Helicobacter pylori*; *M.t., Mycobacterium tuberculosis*; *N.m., Neisseria meningitidis*; *N.g., Neisseria gonorrhoeae*; *S.p., Streptococcus pneumoniae*; *S.py., Streptococcuspyogenes*; *S.a., Staphylococcus aureus*.

#### Repeat sequences promoting transformation

HGT is associated with the risk of allowing entry of alien and potentially harmful DNA from other organisms such as viruses. Even similar and only slightly diverged DNA from other species may be disadvantageous in a new host with separate sets of adaptations and fine-tuned processes. Various strategies have therefore been used in many bacteria to control the entry and persistence of DNA, including ecological isolation such as competence induction by quorum sensing, restriction modification systems and stringent homologous recombination. The *Neisseria* sp. and members of the *Pasteurellaceae*, such as *H. influenzae*, discriminate between homologous and alien DNA by recognizing a short specific sequence in DNA from their own genus. These sequences are known as DNA uptake sequence (DUS) and uptake signal sequence (USS). DUS/USS are found in exceptionally high numbers throughout the genomes of these species, ensuring that almost any piece of the chromosome of a certain length will contain such a signal and hence be recognized, taken up and finally constitute a substrate for homologous recombination (Goodman & Scocca, 1991; Ambur *et al.*, 2007). In contrast, the pneumococci and streptococci use quorum sensing of a competence-stimulating peptide and fratricide to ensure that most of the DNA available in their surroundings is homologous (Johnsborg *et al.*, 2007). Again, the positive effects of allowing HGT has driven the evolution of different strategies that ultimately produce the same result, namely homologous recombination between closely related alleles.

The identification of the positive effects that have influenced the evolution of transformation has not been straightforward, and many selective pressures have been proposed, most of which are not mutually exclusive. Firstly, it has been proposed that transformation evolved from its ability to provide nutrients for the recipient organism, also known as the sex-for-food hypothesis. The rationale behind this notion is that in many organisms, transformation is induced upon starvation and would provide a good system to ensure uptake of high-energy compounds that could promote survival (Redfield *et al.*, 1997; Palchevskiy & Finkel, 2006). Other hypotheses are based on incoming DNA providing a benefit after having been recombined into the chromosome and include, for example, sex for repair and in response to oxidative stress (Nedelcu & Michod, 2003; Nedelcu *et al.*, 2004; Michod *et al.*, 2008) and innovation (Ochman *et al.*, 2000; Narra & Ochman, 2006; Jeon *et al.*, 2008). An in-depth discussion and evaluation of these hypotheses are beyond the scope of this review and the debate on the evolution of transformation and bacterial sex in general is still ongoing (for excellent reviews, please see Johnsborg *et al.*, 2007; Michod *et al.*, 2008). Our own studies of DUS, itself a sign of transformation, have shown that that the complex DUS-mediated control of transformation is not likely to have evolved for its ability to import completely novel sequences. The genomic distribution of these sequences showed that DUS were over-represented in the conserved common core genome, under-represented in regions under diversification, and absent in both recently acquired genes and recently lost core genes (Treangen *et al.*, 2008). Previously, we have found that DUS and USS in *Neisseria* sp. and *H. influenzae*, respectively, are biased toward 3R genes, suggesting that a functional relationship between genome maintenance and transformation exists (Davidsen *et al.*, 2004). In addition, DUS occurrences correlate with the size of conversion fragments. We have therefore proposed that transformation has evolved from its ability to incorporate homologous sequences, either for the regeneration of damaged DNA or from benefits associated with the reassortment of alleles, or both (Treangen *et al.*, 2008). We hypothesize that the core genomes in species that have no means of biasing their DNA uptake, such as *S. pneumoniae, H. pylori* and C. *jejuni*, still could have experienced more recombination during the evolutionary time than the more variable parts of their respective genomes. Although speculative, such frequent nonrandom allelic replacements could be generated by biases at the level of recombination by, for example, repeats. We have no indication that nondiscriminatory transformation differs from the signature-discriminating DUS/USS system of *Neisseria/Pasteurellaceae* in its regenerative properties or that these species experience fundamental differences in DNA damage or the need to reassort alleles. On the contrary, we suspect that these diverse transformation systems have been shaped by the same evolutionary forces. Thus, the study of the genomics of DUS/USS represents an example of convergent evolution (Davidsen *et al.*, 2004), which may also be helpful in generating new testable hypotheses regarding transformation and its history in other organisms. Comparative genomics in a multispecies approach could elaborate on this hypothesis and increase our understanding of the selective advantages of transformation. The hypothesis that transformation evolved due to its ability to provide substrate for recombination has also been strengthened by the observation that these two processes are physically linked in space and time (Kidane & Graumann, 2005).

### Conjugation-related genes: type IV secretion in bacterial DNA transfer/sex

#### The versatile type IV secretion (T4S) systems

T4S systems are involved in the transport of macromolecules such as proteins and DNA across the outer envelope of bacteria. These systems are primarily known from Gram-negative bacteria, where they transport components over both the cytoplasmic and the outer membranes. Interestingly, the conjugation systems that transfer plasmid DNA are one form of the T4S system and are also described for Gram-positive bacteria (Grohmann *et al.*, 2003). While proteins are secreted by the T4S systems and can also be transferred through the plasma membrane of a target cell, DNA can also be secreted and even imported by the T4S system (Ding *et al.*, 2003; Economou *et al.*, 2006). Bacterial conjugation systems represent a prominent subfamily of the T4S systems, while the VirB/D4 system in *A. tumefaciens* is the prototype example of a T4S system. A subclassification of the T4S systems was established based on ancestral lineage, building on two main groups (Christie & Vogel, 2000), followed by a subsequent systematic organization/grouping of all known T4S systems based on their function in conjugation, DNA uptake and release, and effector translocation (Cascales & Christie, 2004). Further analysis of the evolution of the T4S systems based on a protein homology-network defined them into four groups (Medini *et al.*, 2006). Core T4S proteins are part of all T4S systems and can be complemented with independently recruited subunits or proteins to gain a system-specific function (Ding *et al.*, 2003; Medini *et al.*, 2006). It was also suggested that those T4S systems particularly involved in HGT between species led to a functional divergence of these systems (Frank *et al.*, 2005). The secretion of DNA has probably evolved from protein secretion systems (Cascales & Christie, 2003, 2004). The DNA-binding relaxases recognize and translocate DNA, which is suggested to lead to an only coincidental ‘hitch-hiking’ of DNA together with the protein secreted (Cascales & Christie, 2004; Lybarger & Sandkvist, 2004; Chen *et al.*, 2005).

Among the pathogenic bacteria assessed in this review, *C. jejuni, H. pylori, H. influenzae* and *N. meningitidis* as well as *N. gonorrhoeae* possess one or more T4S systems (Table 5) (Hofreuter *et al.*, 1998, 2001; Bacon *et al.*, 2000, 2002; Cascales & Christie, 2004; Snyder *et al.*, 2005). The *C. jejuni* strain 81-176 pTet plasmid is a true conjugative plasmid. It coexists with the smaller pVir plasmid, which also encodes a T4S system, and influences *C. jejuni* virulence (Bacon *et al.*, 2000; Batchelor *et al.*, 2004). Other conjugative plasmids in *C. jejuni* had, as opposed to pVir, no influence on the invasiveness of this bacterium, but often encode antibiotic resistance (Schmidt-Ott *et al.*, 2005; Dasti *et al.*, 2007). Furthermore, other conjugative plasmids are found in other *Campylobacter* species (Boosinger *et al.*, 1990; Fouts *et al.*, 2005).

**Table 5:** The presence and absence of genes related to T4S systems in microbial genomes

		Organism										
Gene	Description	*B.s.*	*E.c*. (IncF)	*C.j*. (Vir)	*H.i*. (Tra)	*H.p*. (Com)	*N.g*. (GGI)	*N.m*. (GGI)	*M.t.*	*S.a.*	*S.p.*	*S.py.*
VirB1					^*^							
VirB2	pilin		^*^		^*^		^*^	^*^				
VirB3			^*^		^*^		^*^	^*^				
VirB4	ATPase		^*^	^*^	^*^	^*^	^*^	^*^				
VirB5			^*^		^*^		^*^	^*^				
VirB6			^*^		^*^		^*^	^*^				
VirB7	lipoprotein		^*^	^*^	^*^	^*^	^*^	^*^				
VirB8	pore protein			^*^	^*^	^*^						
VirB9	pore protein		^*^	^*^	^*^	^*^	^*^	^*^				
VirB10	pore protein		^*^	^*^	^*^	^*^	^*^	^*^				
VirB11	ATPase				^*^	^*^						
VirD4	T4CP		^*^	^*^	^*^		^*^	^*^				

*B.s., Bacillus subtilis*; *E.c., Escherichia coli*; *C.j., Campylobacter jejuni*; *H.i., Haemophilus influenzae*; *H.p., Helicobacter pylori*; *M.t., Mycobacterium tuberculosis*; *N.m., Neisseria meningitidis*; *N.g., Neisseria gonorrhoeae*; *S.p., Streptococcus pneumoniae*; *S.py., Streptococcuspyogenes*; *S.a., Staphylococcus aureus*.

A detailed description of the single components is given by Christie *et al.* (2005).

Another pathogenic member of the order *Campylobacteriales* is *H. pylori*, which has three T4S systems: the Com-system, the Cag- or HP-system and the Tsf3-system (Chen *et al.*, 2005; Zhong *et al.*, 2007). While the Cag- or HP-system is used for exotoxin effector translocation and the function of the Tsf3-system is unknown, the Com-system has evolved for DNA transport (Hofreuter *et al.*, 2001). The conjugation-like Com system is special in that it is used for the uptake of DNA, thus translocating DNA in a direction opposite to that for secretion. This might also be the case for the *C. jejuni* Vir system. These ‘competence’ systems have probably evolved to increase the possibilities for genetic variation or renewal, leading to enhanced cellular fitness, survival and invasion of the eukaryotic host (Ding *et al.*, 2003). In addition, the transfer of chromosomally encoded properties by a conjugation-like mechanism may contribute to horizontal DNA transfer between different members of the *Campylobacteriales* group (Oyarzabal *et al.*, 2007). Little is known about the T4S systems in *H. influenzae*. There are two Tra-like plasmid-encoded systems (Smoot *et al.*, 2002; McGillivary *et al.*, 2005).

#### T4S systems on genomic islands (GIs)

Recently, a GI containing a T4S system, which is evolutionarily distant from the plasmid-based systems and a vector for antibiotic resistance, was discovered (Juhas *et al.*, 2007a, b). This system belongs to a new type of T4S systems found in a wide number of bacteria. They were named GI-like T4S systems and allow GIs encoding many different properties to mobilize and spread (Juhas *et al.*, 2008).

The gonococcal genetic island (GGI) was first identified in *N. gonorrhoeae*. The T4S system encoded by GGI is related to the conjugational F plasmid system of *E. coli* and is used by the bacteria for secretion of chromosomal DNA (Ding *et al.*, 2003; Hamilton *et al.*, 2005). Later, complete and partial forms of the GGI were also found in *N. meningitidis* (Snyder *et al.*, 2005). Approximately 80% of gonococcal strains and some *N. meningitidis* strains carry the GGI, probably inserted by the site-specific recombinase XerCD into the *dif* site (Dillard & Hamilton, 2002; Hamilton *et al.*, 2005). The sequence of the GGI is characterized by a low G+C and low DUS content, suggesting that it is not of neisserial origin, but the amelioration of some regions to a typical neisserial composition indicate an already long-term existence of GGIs in neisserial genomes. As chromosomal DNA is secreted by the T4S system encoded by GGI, no direct contact between the donor and recipient of DNA is needed. This may be so because *Neisseria* species are naturally competent throughout their life cycle and preferentially take up DUS-containing DNA (Lie, 1965; Sparling, 1966; Mathis & Scocca, 1982; Goodman & Scocca, 1991) The GGI contains only one DUS per 10 kb, which is only about 10% of the average DUS density found in the whole genome, but, in addition, it contains several incomplete DUS with one mutation showing that the DUS may be on the way to establish itself in the GGI. For the stable maintenance of GGI in the neisserial genome, the imperfections of one *dif* site were shown to be responsible, because reversion to a perfect site led to significant loss of the GGI (Dillard & Dominguez, 2008). The two parts of the GGI missing in *N. meningitidis* serogroup H and Z strains are flanked by DUS (Snyder *et al.*, 2005). These sites may be the sites of recombination that led to an excision of the sequence blocks (Treangen *et al.*, 2008). Because surrounding sequences of the GGI are still available, they may serve as a target for reintroduction of chunks of DNA by recombination with GGI DNA taken up by the bacterium through the DUS-specific uptake/recombination system. The effects of the GGI are still mostly obscure. It was shown that for the peptidoglycan fragment release in culture, neither the T4S system components nor the GGI-encoded lytic transglycosylases AtlA and LtgX are required for this process, but that the presence of the GGI can bypass the TonB-dependent iron acquisition of intracellular gonococci (Hagen *et al.*, 2006; Cloud-Hansen *et al.*, 2008). On the other hand, the high number of strains of *N. gonorrhoeae* that host a GGI, which can support efficient conjugation, may explain why plasmids, and the consequent antibiotic resistance when selective pressures exert their action, are more prevalent in gonococcal than in meningococcal strains.

## Conclusions

Acquisition and loss of genetic material are essential forces in bacterial microevolution, also challenging functions involved in DNA repair, recombination and HGT. These functions have been repeatedly linked with adaptation of lineages to new lifestyles, and in particular to pathogenicity. Comparative genomics has the potential to elucidate this genetic flux, but there are many methodological challenges involved in inferring gene content and evolutionary events from collections of genome sequences. Here, we have described a method for detecting the presence or the absence of genes in whole genome sequences to elucidate the impact of gene content on microbial lifestyle. Our approach is purely sequence based and relies on gene identification. We have demonstrated its use on datasets from the genomes of *C. jejuni, H. influenzae, H. pylori, M. tuberculosis*, pathogenic *Neisseria, S. pneumoniae, S. pyogenes* and *S. aureus*. In all these examples, we found interesting variations in the presence and absence/gain and loss of genetic material, which correlate with their niches and fitness for survival.

Competence for transformation, according to the gene content detected in many genomes, might be under-rated in many microbial species, including pathogens. At the same time, the different strategies in Gram-negative and Gram-positive organisms to achieve the net result of competence for transformation, leading to the same outcome, namely preferential uptake of its own DNA, represent an exciting diversity in biology. In this context, the presence of repeats, such as DUS and USS, has a tendency to accumulate in the core genome (Treangen *et al.*, 2008), emphasizing their importance. Recently, the linkage between transformation and recombination, including the close proximity of the recombination process to the cytoplasmatic side of the inner membrane (Kidane & Graumann, 2005), has been elucidated. Taken together, this study of the presence and the absence of genes related to DNA metabolism and HGT enlightens how the gene profile affects the lifestyle of microbial pathogens in their respective niches.

## Supporting Information

**Fig. S1.** Sequence conservation of the MutL metal-binding motif DQH/MA(X)_2_E(X)_4_E based on a MutL alignment of entries from 822 organisms.

**Table S1.** Bacteria containing a MutH homolog.
