# Supplementary material for: Genome dynamics in major bacterial pathogens
Source: FEMS Microbiol Rev. 2009 Apr;33(3):453–70. doi: 10.1111/j.1574-6976.2009.00173.x (PMC2734928; doi:10.1111/j.1574-6976.2009.00173.x)
Supplement: Supplementary file 1 [file fmr0033-0453-SD1.doc]

**Supporting Information**

**Fig. S1.**

Sequence conservation of the MutL metal binding motif DQH/MA(X)_2_E(X)_4_E based on a MutL alignment of entries from 822 organisms. The motif image was generated with Weblogo

(Croo*ks, et a*l., 2004)

| **Organism** | **Phylum** | **Class** | **Order** | **Family** | **Genus** |
| --- | --- | --- | --- | --- | --- |
| *Aeromonas hydrophila* | Proteobacteria | Gammaproteobacteria | Aeromonadales | Aeromonadaceae | Aeromonas |
| *Aeromonas salmonicida* | Proteobacteria | Gammaproteobacteria | Aeromonadales | Aeromonadaceae | Aeromonas |
| *Pseudoalteromonas haloplanktis* | Proteobacteria | Gammaproteobacteria | Alteromonadales | Pseudoalteromonadaceae | Pseudoalteromonas |
| *Psychromonas ingrahamii* | Proteobacteria | Gammaproteobacteria | Alteromonadales | Psychromonadaceae | Psychromonas |
| *Shewanella sp.* | Proteobacteria | Gammaproteobacteria | Alteromonadales | Shewanellaceae | Shewanella |
| *Colwellia psychrerythraea* | Proteobacteria | Gammaproteobacteria | Alteromonadales | Colwelliaceae | Colwellia |
| *Idiomarina loihiensis* | Proteobacteria | Gammaproteobacteria | Alteromonadales | Idiomarinaceae | Idiomarina |
| *Pseudoalteromonas atlantica* | Proteobacteria | Gammaproteobacteria | Alteromonadales | Pseudoalteromonadaceae | Pseudoalteromonas |
| *Nitrosococcus oceani* | Proteobacteria | Gammaproteobacteria | Chromatiales | Chromatiaceae | Nitrosococcus |
| *Citrobacter koseri* | Proteobacteria | Gammaproteobacteria | Enterobacteriales | Enterobacteriaceae | Citrobacter |
| *Enterobacter sakazakii* | Proteobacteria | Gammaproteobacteria | Enterobacteriales | Enterobacteriaceae | Cronobacter |
| *Enterobacter sp.* | Proteobacteria | Gammaproteobacteria | Enterobacteriales | Enterobacteriaceae | Enterobacter |
| *Escherichia coli* | Proteobacteria | Gammaproteobacteria | Enterobacteriales | Enterobacteriaceae | Escherichia |
| *Klebsiella pneumoniae* | Proteobacteria | Gammaproteobacteria | Enterobacteriales | Enterobacteriaceae | Klebsiella |
| *Pectobacterium atrosepticum* | Proteobacteria | Gammaproteobacteria | Enterobacteriales | Enterobacteriaceae | Pectobacterium |
| *Photorhabdus luminescens* | Proteobacteria | Gammaproteobacteria | Enterobacteriales | Enterobacteriaceae | Photorhabdus |
| *Salmonella sp.* | Proteobacteria | Gammaproteobacteria | Enterobacteriales | Enterobacteriaceae | Salmonella |
| *Serratia sp.* | Proteobacteria | Gammaproteobacteria | Enterobacteriales | Enterobacteriaceae | Serratia |
| *Shigella sp.* | Proteobacteria | Gammaproteobacteria | Enterobacteriales | Enterobacteriaceae | Shigella |
| *Sodalis glossinidius* | Proteobacteria | Gammaproteobacteria | Enterobacteriales | Enterobacteriaceae | Sodalis |
| *Yersinia sp* | Proteobacteria | Gammaproteobacteria | Enterobacteriales | Enterobacteriaceae | Yersinia |
| *Buchnera aphidicola* | Proteobacteria | Gammaproteobacteria | Enterobacteriales | Enterobacteriaceae | Buchnera |
| *Legionella pneumophila* | Proteobacteria | Gammaproteobacteria | Legionellales | Legionellaceae | Legionella |
| *Sorangium cellulosum* | Proteobacteria | Deltaproteobacteria | Myxococcales | Polyangiaceae | Sorangium |
| *Actinobacillus sp.* | Proteobacteria | Gammaproteobacteria | Pasteurellales | Pasteurellaceae | Actinobacillus |
| *Haemophilus sp.* | Proteobacteria | Gammaproteobacteria | Pasteurellales | Pasteurellaceae | Haemophilus |
| *Mannheimia succiniciproducens* | Proteobacteria | Gammaproteobacteria | Pasteurellales | Pasteurellaceae | Mannheimia |
| *Pasteurella multocida* | Proteobacteria | Gammaproteobacteria | Pasteurellales | Pasteurellaceae | Pasteurella |
| *Baumannia cicadellinicola* | Proteobacteria | Gammaproteobacteria | unclassified | Candidatus Baumannia |  |
| *Photobacterium profundum* | Proteobacteria | Gammaproteobacteria | Vibrionales | Vibrionaceae | Photobacterium |
| *Vibrio sp.* | Proteobacteria | Gammaproteobacteria | Vibrionales | Vibrionaceae | Vibrio |

**Table S1.**. Bacteria containing a MutH homolog
